# Nutritional implications of olives and sugar: attenuation of post-prandial glucose spikes in healthy volunteers by inhibition of sucrose hydrolysis and glucose transport by oleuropein

**DOI:** 10.1007/s00394-018-1662-9

**Published:** 2018-03-09

**Authors:** Asimina Kerimi, Hilda Nyambe-Silavwe, Alison Pyner, Ebun Oladele, Julia S. Gauer, Yala Stevens, Gary Williamson

**Affiliations:** 1 School of Food Science and Nutrition, University of Leeds, Leeds, LS2 9JT UK; 2 BioActor, Oxfordlaan 70, 6229 EV Maastricht, The Netherlands

**Keywords:** Olives, Sugar, Post-prandial, Oleuropein, Transport, Sucrase

## Abstract

**Purpose:**

The secoiridoid oleuropein, as found in olives and olive leaves, modulates some biomarkers of diabetes risk in vivo. A possible mechanism may be to attenuate sugar digestion and absorption.

**Methods:**

We explored the potential of oleuropein, prepared from olive leaves in a water soluble form (OLE), to inhibit digestive enzymes (α-amylase, maltase, sucrase), and lower [^14^C(U)]-glucose uptake in *Xenopus* oocytes expressing human GLUT2 and [^14^C(U)]-glucose transport across differentiated Caco-2 cell monolayers. We conducted 7 separate crossover, controlled, randomised intervention studies on healthy volunteers (double-blinded and placebo-controlled for the OLE supplement) to assess the effect of OLE on post-prandial blood glucose after consumption of bread, glucose or sucrose.

**Results:**

OLE inhibited intestinal maltase, human sucrase, glucose transport across Caco-2 monolayers, and uptake of glucose by GLUT2 in *Xenopus* oocytes, but was a weak inhibitor of human α-amylase. OLE, in capsules, in solution or as naturally present in olives, did not affect post-prandial glucose derived from bread, while OLE in solution attenuated post-prandial blood glucose after consumption of 25 g sucrose, but had no effect when consumed with 50 g of sucrose or glucose.

**Conclusion:**

The combined inhibition of sucrase activity and of glucose transport observed in vitro was sufficient to modify digestion of low doses of sucrose in healthy volunteers. In comparison, the weak inhibition of α-amylase by OLE was not enough to modify blood sugar when consumed with a starch-rich food, suggesting that a threshold potency is required for inhibition of digestive enzymes in order to translate into in vivo effects.

## Introduction

**Fig. 1:**
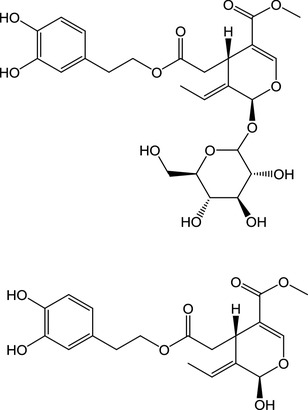
Chemical structures. Structure of oleuropein (IUPAC name: methyl (4S,5E,6S)-4-[2-[2-(3,4-dihydroxyphenyl)ethoxy]-2-oxoethyl]-5-ethylidene-6-[(2S,3R,4S,5S,6R)-3,4,5-trihydroxy-6-(hydroxymethyl)oxan-2-yl]oxy-4H-pyran-3-carboxylate) and “oleuropein aglycone” (IUPAC name: methyl (4S,5E,6R)-4-[2-[2-(3,4-dihydroxyphenyl)ethoxy]-2-oxoethyl]-5-ethylidene-6-hydroxy-4H-pyran-3-carboxylate)

Intervention and epidemiological studies have highlighted the importance of olives and olive oil in the Mediterranean diet. In the large intervention study “Predimed”, volunteers receiving a Mediterranean diet supplemented with extra-virgin olive oil for 4.8 years relative to a control diet (with advice to reduce fat) exhibited a lower incidence of cardiovascular events [1]. Increased olive oil consumption was associated with a dose-dependent reduction in cardiovascular disease mortality, with the highest quartile benefitting from a 44% reduction in risk [2]. In addition to the monounsaturated lipid components of olives and olive oil, one of the main biologically active substances is oleuropein (Fig. 1), which is found at high levels in olives (up to 70 mg/100 g), at much higher levels in unprocessed olives [3], and at lower levels in olive oil [4]. Oleuropein is also a major constituent of olive leaves, and “olive leaf extract” (OLE) is available as a supplement, in which the main component is oleuropein. Numerous studies on rodents have shown that oleuropein has a beneficial effect on various factors related to diabetes and cardiovascular development [5–8]. In humans, a randomised, double-blind, placebo-controlled crossover single dose study showed that OLE significantly attenuated the digital volume pulse-stiffness index in healthy volunteers [9]. However, another randomised, double-blind, placebo-controlled crossover study using 500 mg OLE together with 100 mg green coffee bean extract and 150 mg beetroot powder for 6 weeks showed no effect on blood pressure, blood lipids, glucose or insulin [10]. In a randomised placebo-controlled trial, adults with type 2 diabetes showed lowered HbA1c and fasting plasma insulin after 14 weeks of 500 mg OLE [11].

The mechanism by which oleuropein might affect diabetes risk and development of related cardio-metabolic complications is not clear. Since one of the suggested actions of green tea polyphenols against diabetes is to improve glucose homeostasis [12] and attenuate post-prandial glucose absorption and metabolism [13, 14], we explored the possibility that oleuropein could work at least in part in a similar way, by attenuating carbohydrate digestion. Preliminary evidence has shown that oleuropein might inhibit α-glucosidase activities. However, some of the conducted studies used the enzyme from yeast (*Saccharomyces cerevisiae*), which has very little homology to the target human enzyme, and shows a completely different profile of inhibition. For example, the drug acarbose inhibited rat and human maltase activities with IC_50_ of 0.42 and 5.7 μM respectively [15], but inhibited yeast α-glucosidase activity much less effectively with IC_50_ ~ 250 μM [16]. When using the yeast enzyme, hydroxytyrosol and oleuropein both showed inhibition [16], and some extracts from olive oil also inhibited the yeast enzyme [17]. For porcine α-amylase, oleuropein and hydroxytyrosol were both weak inhibitors [16]. When a large dose of OLE was given to healthy volunteers together with a large dose of rice, there was no difference in postprandial blood glucose in healthy volunteers, but a decrease was observed in “borderline” volunteers (although “borderline” was not defined). The authors ascribed these changes to inhibition of human salivary and pancreatic α-amylase by luteolin glucoside and oleanolic acid [18]. As the results in the literature are mixed, with very little information on inhibition of human enzymes which constitute the most relevant in vitro system, we have evaluated the effect of OLE on both carbohydrate digestion enzymes and sugar transporters in vitro, and demonstrated how these mechanisms translate into modulation of post-prandial glucose in vivo in healthy volunteers consuming different types of carbohydrate.

## Materials and methods

### Materials

OLE in the form of capsules (Bonolive^®^, Olecol^®^) and as powder was obtained from BioActor B.V. (Maastricht, The Netherlands). d-Glucose and d-fructose were obtained from Fisher Scientific (Loughborough, UK). Acetone-protein extract from rat intestine, pancreatin from porcine pancreas, pepsin, Dulbeccos’s modified Eagle’s Medium (DMEM), foetal bovine serum (FBS), glucose and sucrose for in vitro studies, non-essential amino acids, penicillin/streptomycin solution, trypsin, hexokinase reagent, potassium oxalate/sodium fluoride tubes, acarbose, oleuropein (> 98%), dimethyl sulfoxide (DMSO), amylose from potatoes, maltose and chromatographically purified human salivary α-amylase type IX-A were purchased from Sigma–Aldrich (Dorset, UK). Caco-2/TC7 cells were a kind gift from Dr M. Rousset, (U178 INSERM, Villejuif, France). D-[^14^C(U)]-glucose was from Perkin Elmer (Boston, USA) and D-[^14^C(U)]-fructose was from Hartmann Analytic (Braunschweig, Germany). Milli-Q water was used to make up all laboratory solutions (Millipore, Watford, UK). Glucose powder for human studies was purchased from Greens Pharmacy (Amazon, UK), and sucrose for consumption was purchased from a local supermarket. Bread was 109 g Warburtons™ medium sliced white bread that contained 50 g of available carbohydrate [19]. Accu-Chek Aviva (Roche Scientific) glucometers and test strips were obtained from Boots (Nottingham, UK). Accu-Chek glucose control solutions were obtained from Weldricks Pharmacy (Doncaster, UK).

### Analysis of oleuropein in olives and in supplements

Samples were analysed for oleuropein content using an Agilent Technologies 1200 series HPLC with diode array detection and a ZORBAX Eclipse plus C18 column (2.1 mm × 100 mm, 1.8 μm; Agilent Technologies) kept at 35 °C. The injection volume was 5 μl. Separation was achieved on a gradient of solvent A (0.2% acetic acid, 99.8% water) and solvent B (acetonitrile) at a flow rate of 0.25 ml/min, starting at 10% B and at 9 min 35% B, at 10 min 100% B until 13 min and returning at 14 min to 10% B. For quantitation of oleuropein, an external standard curve was constructed. Oleuropein was dissolved in 100% DMSO (40 mM) and diluted in the starting conditions of the chromatographic gradient (10% acetonitrile, 0.2% acetic acid, 89.8% water).

For analysis of the OLE samples, 2.5 mg of powder was dissolved in 4 ml water, and the solution filtered through a 0.2 μm PTFE filter. Samples were analysed three times in duplicate. For analysis of olives, ~ 2.5 g of olives were cut into small pieces and homogenised with 3 ml of 80% methanol. The solution was centrifuged, the pellet re-extracted with an additional 3 ml methanol, the supernatants pooled and filtered through a 0.2 μm PTFE filter. Olive samples were also spiked with different amounts of oleuropein at the homogenisation stage to calculate the extraction efficiency, which was 72 ± 6%. The oleuropein content in olives is shown in Table 1.

**Table 1:** Composition of OLE preparations and olives used in these studies

	Oleuropein content
Bonolive^®^ powder^1^	41.8 ± 0.9% w/w
Olecol^®^ powder	19.8 ± 0.5% w/w
Kalamata olives	34.8 ± 0.4 mg oleuropein/100 g

Data are mean and standard deviation of *n* = 3 determinations

^1^Bonolive^®^ powder also contained tyrosol (0.045% w/w) and hydroxytyrosol (0.0065% w/w) as estimated by HPLC (see “Materials and methods”)

Bonolive^®^ is a standardised water soluble extract prepared from the cut leaves of *Olea europaea* L. (the common olive tree), and has been used in previous interventions, such as Filip et al., where it was safely fed to volunteers for 12 months [20]. The total phenolic content of Bonolive^®^ was 50%, and included inert excipients added for encapsulation. Of the phenolic content, oleuropein constituted ~ 80%, equivalent to 40% content of oleuropein with no other phenolic compounds exceeding ~ 3%. The studies reported here were on OLE in the form of Bonolive^®^, apart from intervention studies 1 and 2 on OLE in capsules which were performed on Olecol^®^, which is also water-soluble, and had an oleuropein content of ~ 50% of that of Bonolive^®^ (Table 1).

### Hydrolysis and stability of oleuropein

Enzyme solutions were prepared by addition of 300 mg of porcine pancreatin, acetone extract of rat intestine, or hesperidinase to 12 ml sodium phosphate buffer (pH 6). OLE (4.58 mg/ ml) and quercetin-3-*O*-glucoside (0.1 mM) were dissolved in water. After incubation for the indicated time under the specified conditions, reactions were stopped by placing in a boiling water bath for 5 min, followed by 2 min on ice, before 1.1 ml acetonitrile and 9.1 ml water were added and the samples filtered through a 0.2 μm PTFE filter for analysis by HPLC. The final protein concentration in the oleuropein enzymic solution was: 0.079 mg/ml for pancreatin, 0.124 mg/ml for the rat intestinal extract and 0.0013 mg/ml for hesperidinase. For quercetin-3-*O*-glucoside, enzymic solutions were 0.091, 0.141 and 0.0015 mg/ml respectively. Quercetin-3-*O*-glucoside was analysed using the same gradient as described above using an external calibration curve.

### Measurement of human salivary α-amylase activity

Inhibition of α-amylase was measured as described previously, using human salivary α-amylase with an optimised protocol [21].

### Measurement of maltase and sucrase activities

Inhibition of sucrase and maltase was tested using a protein extract of rat intestine and Caco-2/TC7 cells as the enzyme source (cultured as described below) according to a previously optimized protocol, using sucrose and maltose as substrates respectively [15]. Where indicated, oleuropein was removed by SPE prior to glucose quantification [15].

### Purification of maltase from rat intestine

A protein extract from rat intestine was prepared freshly before use by acetone precipitation, and used as the enzymic source of α-glucosidase. The powdered extract (30 mg) was added to 1 ml of water, vortexed for 10 s, centrifuged (15 min, 10,000×*g*, 4 °C) and the supernatant collected. Papain (in 0.012 M EDTA, 0.012 M cysteine, 0.024 M potassium phosphate buffer) was added to the acetone powder (0.1% w/w) to remove the enzyme from its membrane attachment [22]. Following incubation for 30 min at 37 °C the sample was placed on ice and centrifuged (105,000*g*, 4 °C) for 60 min. Papain addition increased the apparent total enzyme activity by ~ 36%. Enzyme purification was carried out with an ÄKTA Purifier System (GE Healthcare, Fairfield, CT, USA) controlled by a PC running GE Unicorn software (5.11). The papain-treated product (1 ml) was loaded manually using a syringe and a sample loop of 0.1 ml on a Q Sepharose Fast Flow XK 16/40 column (GE Healthcare, USA) and eluted with a gradient of sodium phosphate buffer (10 mM, pH 7, Solvent A) and 0.2 M KCl (Solvent B) at a flow rate of 0.2 ml/min. The elution was followed at 280 nm. Maltase eluted at the beginning of the KCl gradient and fractions were collected with a Frac-900 fraction collector in 18 ml tubes. Fractions from multiple runs were pooled and freeze dried to obtain sufficient enzyme for the inhibition assays. The procedure resulted in a 2300-fold purer enzyme based on specific maltase activity.

### Cell culture

Caco-2/TC7 cells were cultured in DMEM (25 mM glucose) supplemented with 20% (v/v) foetal bovine serum, 2% (v/v) Glutamax (Invitrogen, Thermo Fisher Scientific, UK), 2% (v/v) non-essential amino acids, 100 U/mL of penicillin and 0.1 mg/mL streptomycin. Cells were seeded at a density of 1.2 × 10^6^ cells per 75 cm^2^ culture flask (37 °C, 10% CO_2_). For enzyme assays, cells allowed to differentiate for 21–23 days were scraped and pellets were snap frozen in a dry ice-ethanol bath and kept at − 80 °C.

### Sucrose transport across differentiated Caco-2/TC7 cell monolayers

Cells were cultured on Transwell plates (Corning 3412, Appleton Woods, UK) as previously described [14]. Caco-2/TC7 cells were incubated with 1, 5 or 25 mM sucrose added in the apical compartment for 60 min and the concentration of glucose and fructose were measured in the apical and basolateral solutions. OLE (in the form of powder extract Bonolive^®^) was added to the apical solution at 0.5, 1.5, or 3.0 mg/mL resulting in 0.2, 0.6, 1.2 mg oleuropein/ml respectively.

### High performance anion exchange chromatography with pulsed amperometric detection (HPAE-PAD)

Analysis of sugars was conducted on an ICS-4000 ion capillary system equipped with a pulsed amperometric detector (PAD), an electrolytic eluent generator to automatically produce an isocratic potassium hydroxide eluent and an AS-AP autosampler (Dionex, Thermo Fisher Scientific, Hemel Hempstead, UK). Instrument control, data collection and processing was carried out through a Chromeleon software (version 7.2 SR4, Thermo Fisher Scientific, UK). A palladium reference electrode and a gold working electrode were used with a collection rate of 2.00 Hz using the “Gold, Carbo, Quad” waveform. Separation of sugars was achieved at a flow rate of 0.008 ml/min on a Carbopac PA20 column (0.4 × 150 mm) attached to a CarboPac PA20 Guard (0.4 × 35 mm) (Thermo Fisher Scientific, UK) kept at 30 °C (0.04 µl injection volume). Quantitation of sugars was based on an external calibration curve as previously described [15]. Apical and basolateral samples from transport studies were diluted fivefold with Milli-Q water; the limit of quantification of glucose and fructose was 1 μM, and so samples with a concentration > 5 μM could be quantified. The presence of OLE in the apical solutions interfered with measurements of sugars and therefore separate standard curves were constructed incorporating final concentrations of 0.04, 0.12 and 0.24 mg oleuropein/ml. All standards and samples were filtered with 0.2 µM PTFE filters and kept at 4 °C in the autosampler until analysis.

### Glucose transport across differentiated Caco-2/TC7 cell monolayers

Glucose transport was assessed in Caco-2/TC7 fully differentiated cell monolayers cultured and maintained for experiments as previously described on Transwell plates (Corning 3412, Appleton Woods, UK) for 23 days [14]. For the glucose transport assay from the apical to the basolateral side, [^14^C(U)]-glucose (0.1 μCi/ well) was added to the apical compartment in transport buffer (TBS, 20 mM HEPES, 137 mM NaCl, 4.7 mM KCl, 1.8 mM CaCl_2_) containing either 2, 5, 11 or 25 mM glucose. OLE (in the form of powder extract Bonolive^®^) was prepared in TBS. For transport experiments in the absence of sodium (− Na^+^), TBS was prepared only with KCl. Cell monolayers were incubated for 30 min (37 °C, 5% CO_2_) and solutions from both apical and basolateral compartments were collected for scintillation counting as described before [14].

### Inhibition of transport by GLUT2 and GLUT5 expressed in *Xenopus* oocytes

GLUT2 and GLUT5 were expressed in *Xenopus* oocytes and [^14^C(U)]-glucose and [^14^C(U)]-fructose sugar uptake experiments were performed as previously described [14].

### Characterisation of OLE capsule dissolution under gastrointestinal conditions

In order to mimic the conditions in the stomach, pepsin (3 ml of 1.6 g pepsin powder in 10 ml of 0.1 M HCl) was added to 150 ml water and the pH adjusted to 2.0 using HCl (0.1 M). One OLE capsule (Olecol^®^) was added to the pepsin solution with continuous stirring at 37 °C and dissolution was observed visually.

### Intervention studies on healthy volunteers

All intervention studies were approved by the University of Leeds, Faculties of Mathematics and Physical Sciences and Engineering Ethics Committee (MEEC), were registered at clinicaltrials.gov (approval numbers and clinicaltrials.gov reference numbers shown in Table 2) and were conducted between July 2015 and August 2017. A pre-study questionnaire was used to assess the suitability of participants for the studies. The inclusion criteria were apparently healthy, aged between 18 and 75 years old, not smoking, not diabetic, not on long term prescribed medication, not allergic to olives, not pregnant, not lactating and not on any special diets including weight loss diets or fruit supplements, with fasting blood glucose between 3.9 and 5.9 mM. Written informed consent was provided by all participants before the commencement of the study. Height and weight of volunteers was recorded at the beginning of the study and subjects received the treatments in a cross-over design in randomised order. Control samples were consumed as indicated per study, but the nature of the test food meant that only some studies could be double-blinded as described below. During the studies, participants consumed their normal diet, but were instructed to eat the same evening meal the day before each visit. Participants were asked to arrive at the School of Food Science and Nutrition in the morning around 9.00 am after an overnight fast of 10–12 h. Each participant was assigned a code and all data were stored anonymised. Each visit lasted ~ 3 h and capillary blood samples were obtained by finger prick at 0 min (fasting blood glucose) using an Accu-Chek Aviva glucometer according to the FAO/WHO approved method [23]. The accuracy of the glucometer was tested using control solutions; three separate readings of a 2.5 and a 16.5 mM glucose solution gave 2.5, 2.4 and 2.4 mM; and 16.5, 16.3 and 16.3 mM respectively. Glucometer readings were not significantly different when compared to measurements with the hexokinase-linked assay on the same blood samples (data not shown).

**Table 2:** Details of human intervention studies conducted

Study number	1 and 2	3	4	5	6	7	8
Number of participants	24	16	10	10	10	10	10
Study design	Double-blinded, randomised, crossover, placebo controlled	Randomised, crossover, controlled	Randomised, crossover, controlled	Randomised, crossover, controlled	Randomised, crossover, controlled	Randomised, crossover, controlled	Randomised, crossover, controlled
Gender	11 m; 13 f	5 m; 11 f	3 m; 7 f	7 m; 3 f	1 m; 9 f	4 m; 6 f	1 m; 9 f
Age	23.4 ± 1.4	33.6 ± 6.9	22.9 ± 0.9	23.5 ± 1.0	25.1 ± 1.4	23.6 ± 1.36	23.6 ± 1.5
BMI kg/m^2^	22.7 ± 3.0	24.7 ± 3.9	21.6 ± 2.6	21.5 ± 2.0	22.4 ± 3.0	20.2 ± 1.77	20.1 ± 1.9
Carbohydrate source	Bread (109 g containing 50 g carbohydrate)	Bread (109 g containing 50 g carbohydrate)	Bread (109 g containing 50 g carbohydrate)	Wholemeal bread (132 g containing 50 g carbohydrate)	Glucose (50 g)	Sucrose (50 g)	Sucrose (25 g)
Oleuropein source	OLE in capsules ((1) 500 mg (2) 1000 mg) with 250 ml water	Kalamata olives (100 g) with 200 ml water	OLE (125 mg) dissolved in 200 ml water	OLE (125 mg) dissolved in 200 ml water	OLE (0.125 g) dissolved in 200 ml water	OLE (0.125 g) dissolved in 200 ml water	OLE (0.4 g) dissolved in 250 ml water
Dose of oleuropein (mg)	(1) 100 (2) 200	35	50	50	50	50	160
Number of repeat visits for each arm	1	2	2	2	2	2	2
Ethics approval	MEEC 14-029	MEEC 12-037a	MEEC 15-044	MEEC 15-044	MEEC 15-044	MEEC 15-044	MEEC 15-044
Clinicaltrials.gov	NCT02486978	NCT02669693	NCT03093753	NCT03093753	NCT03093753	NCT03093753	NCT03093753

Volunteers were calm for several minutes before measurements, with the hand palm-side up, and after using a sterile wipe to clean the fingertip, a new lancet was used to puncture the centre of the fingertip with mild pressure to aid blood flow. The first drop of blood was discarded using a cotton swab, and the second drop was used for measurements, which were taken at nine time points—0, 15, 30, 45, 60, 90, 120, 150 and 180 min, and the data used to plot a glucose response curve. The incremental area under the curve (IAUC) was used as the main indicator of changes in glucose response between interventions, and this analysis is reported to be the best method for this type of study [24]. Full details have been described previously [25].

### Specific aspects of the protocol followed for study 1 and 2 on OLE in capsules

Studies with OLE capsules were randomised (Latin square design), double-blinded and placebo controlled. Both study scientists and participants were blinded to the treatment until the end of the study. Participants (*n* = 24) consumed either a placebo (a capsule containing 500 mg cellulose) or a low dose of OLE (one capsule containing 500 mg of OLE, equivalent to 100 mg oleuropein) or twice the same dose of OLE (two capsules totalling 1000 mg of OLE, equivalent to 200 mg oleuropein). The OLE used in the capsules was Olecol^®^. Five minutes after the capsules were consumed, participants were given 109 g of Warburton’s Medium White bread, containing 50 g of available carbohydrates [19]. Timing began when the participant took the first bite of bread, and the time taken to eat the bread was recorded. Each participant was given a total of 250 ml water with each intervention.

### Specific aspects of the protocol followed for study 3 on olives

Sixteen volunteers were recruited. All study participants consumed the test meal (109 g white bread) with or without 100 g olives (Greek Kalamata black olives, Sainsbury’s, UK) in a randomised, crossover design. Olives were de-pitted, cut finely and spread onto the bread, and both test meal and control were consumed with water (200 mL). Participants attended twice for each treatment.

### Specific aspects of the protocol followed for studies 4–8

Each study was randomised with a crossover design. Ten healthy participants were recruited for each study and each attended four visits (two visits per treatment). For studies 4–7, 125 mg of OLE (equivalent to 50 mg oleuropein) was dissolved in 200 ml of water; and for study 8, 0.4 g of OLE (equivalent to 160 mg oleuropein) was dissolved in 250 ml of water (Table 2). Participants consumed the test and control meals in a randomised order and blood samples were collected starting after the first bite or sip. For study 5, Hovis Medium Wholemeal Loaf (800 g) was purchased from the local supermarket and provided 37.8 g carbohydrate per 100 g.

### Statistics and replicates

Intervention studies were carried out with a minimum of 10 participants, which is the recommended minimum number for these types of determinations [23]. For the study on olives, the trial was designed to have 90% power to detect a clinical difference of 15% IAUC between the test and reference meal (*α* = 0.05), where a minimum of 15 volunteers were required for the reference and test meals to achieve the above power and clinical difference. The trapezoidal rule was used to calculate the incremental area under the glucose curves (IAUC) for each volunteer, and data analysis was performed by the two-tailed paired *t* test. Comparisons between control and treatment in [^14^C(U)]-glucose transport experiments were carried out by the two-tailed independent samples *t* test and data are presented as mean ± SD with a minimum of 3 independent experiments and 6 replicates well/experiment. For [^14^C(U)]-glucose and [^14^C(U)]-fructose uptake experiments into *Xenopus* oocytes expressing human GLUT2 and GLUT5, data were normalized against water-injected oocytes for each condition, and the two-tailed independent samples *t* test was used to assess significance.

For sucrase and maltase assays, the percentage inhibition was determined by the following formula: % inhibition = [([Glucose]_Control_-[Glucose]_inhibitor_)/[Glucose]_control_] × 100, where the control is without inhibitor. Statistics were performed by one-way ANOVA using SPSS Statistics version 24 and statistical significance was determined by Tukey–Kramer multiple comparison test (*p* ≤ 0.05). The data are presented as mean ± SEM with minimum of *n* = 3. Cell lysates were prepared from three biological passages of cells and used for IC_50_ determinations. For transport studies, results were normalised to the control and one-way ANOVA performed with statistical significance determined by the Tukey–Kramer multiple comparison test (*p* ≤ 0.05). Three independent transport experiments were performed with three biological passages of cells with six technical replicates for each experiment.

## Results

### OLE composition and stability

**Fig. 2:**
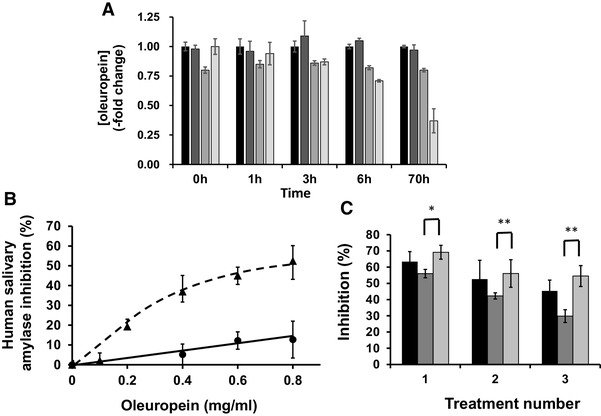
Stability of OLE and interaction with human salivary α-amylase. **a** Enzymatic hydrolysis of oleuropein as assessed using HPLC with diode array detection (see “Materials and methods”). Oleuropein was incubated at 37 °C with porcine pancreatin (79 μg protein/ml) (dark grey bars), a protein extract from rat intestine (124 μg protein/ml) (grey bars), or “hesperidinase” from *A. niger* (1.3 μg protein/ml) (light grey bars), in 0.05 M sodium phosphate buffer pH 6. Black bars indicate oleuropein with no added enzymes. Under similar conditions, quercetin-3-*O*-glucoside (100 μM), as control, was rapidly hydrolysed by the rat intestinal preparation (~ 30% remaining at 3 h, none detectable at 6 h) and by hesperidinase (~ 40% remaining after 6 h, none detectable at 70 h). Quercetin-3-*O*-glucoside was unaffected by pancreatin. **b** Inhibition of human salivary α-amylase using either amylose (black triangle) or amylopectin (black circle) as substrate, and measuring product using 2,4-dinitroasalicylic acid. **c** Effect of combining oleuropein with acarbose on inhibition of human salivary α-amylase. Black bars show OLE alone, dark grey bars show acarbose and the light grey bars show a combination: Treatment number 1: OLE (oleuropein, 0.8 mg/ml), acarbose, 2.5 µM; 2: OLE (oleuropein, 0.6 mg/ml), acarbose, 1.88 µM; 3: OLE (oleuropein, 0.4 mg/ml), acarbose, 1.25 µM. *p ≤ 0.05, **p ≤ 0.01

The olive leaf extract (OLE) tested throughout the studies presented here provided a convenient source of oleuropein in a formulation with enhanced solubility, suitable for both in vitro experiments and in vivo intervention studies. The two preparations used were characterised by HLPC (Table 1). The bioactivities tested here mostly take place in vivo in the gut lumen and intestinal tissues. Once ingested, food components are subjected to digestive enzymes and the pH conditions prevailing in the different sections of the gut lumen. To test if oleuropein would be hydrolysed by digestive enzymes, we carried out in vitro incubations. Neither pancreatic enzymes nor a protein extract from rat intestine hydrolysed oleuropein over an extended period of time (Fig. 2a). As a positive control, we showed that quercetin-3-*O*-glucoside was hydrolysed by the intestinal extract (owing to its content of β-glucosidases [26]) but not by the pancreatic extract, as expected. Under our conditions, both oleuropein and quercetin-3-*O*-glucoside were hydrolysed by the fungal hemicellulase (“hesperidinase” from *A. niger*) preparation [27], but this enzyme is not present in the small intestine. The results imply that oleuropein would exist in the small intestine in its intact form, and could potentially interact with digestive enzymes and transporters.

### Effect of OLE on human salivary α-amylase

OLE inhibited human salivary α-amylase, and the extent of inhibition depended on the substrate (Fig. 2b). When amylopectin was used as substrate, oleuropein showed almost no inhibition, while with amylose the IC_50_ value was ~ 0.8 mg/ml (Fig. 2b; Table 3). Acarbose, as expected, was a potent inhibitor of α-amylase, but in combination with OLE, an additive effect was evident, however no synergy was observed (Fig. 2c).

**Table 3:** Inhibition of digestive enzymes by OLE

Human α-amylase^a^	Human α-amylase^b^	Rat maltase	Human maltase	Rat sucrase	Human sucrase
IC_10_ (mg/ml)^c^	IC_50_ (mg/ml)^c^				
0.8 ± 0.2	0.8 ± 0.1	0.24 ± 0.08	0.52 ± 0.08	ND^d^	1.28 ± 0.4

Data are mean ± SD (*n* = 3)

^a^Amylopectin as substrate at 0.37 mg/ml

^b^Amylose as substrate at 1.0 mg/ml

^c^Values in mg oleuropein /ml

^d^ND = inhibition did not reach 50% at concentrations up to 2 mg oleuropein/ml

### Effect of OLE on α-glucosidase activities

**Fig. 3:**
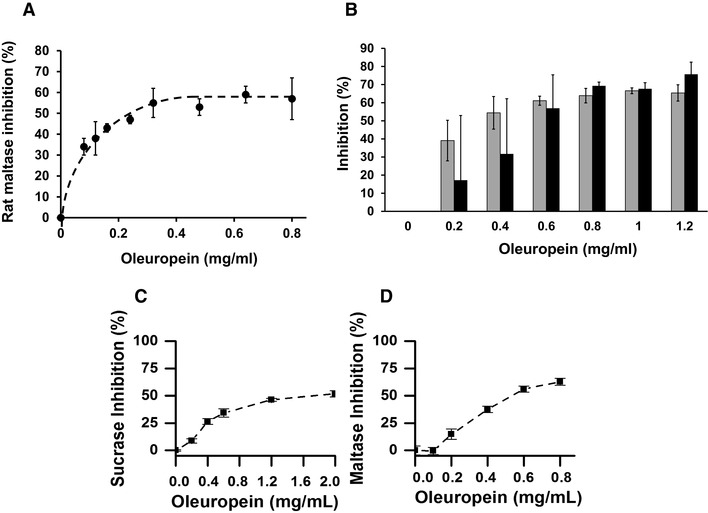
Inhibition of α-glucosidases by OLE. **a** Inhibition of rat intestinal maltase activity, using maltose (4 mM) as substrate, and quantifying glucose produced using a hexokinase-linked assay. **b** Inhibition of crude and purified rat intestinal maltase by OLE. Crude rat intestinal preparation (grey bars) and 2300-fold purified preparation of rat maltase (black bars) were incubated with maltose and inhibitor as described in the experimental section. The IC_50_ values for OLE inhibition were not significantly different (0.46 ± 0.14 and 0.40 ± 0.26 mg/ml of oleuropein respectively). **c, d** Inhibition of human maltase and sucrase activity using Caco-2/TC7 cells as the enzyme source. Glucose was quantified using a hexokinase-linked assay after the removal of interfering compounds by SPE. The IC_50_ values for maltase and sucrase were 1.28 ± 0.4 and 3.2 ± 1.0 mg/mL oleuropein, respectively

OLE inhibited rat intestinal maltase activity (Fig. 3a). Since maltase activity can arise both from maltase-glucoamylase and sucrase-isomaltase enzymes, and the presence of other proteins in the intestinal extract could potentially affect the inhibition, we purified the main maltase activity from rat intestine. Papain digestion was used to free the enzyme from the brush border of the enterocytes, and this treatment resulted in an apparent 36% increase in activity. The enzyme was purified 2300-fold. Only a small amount of enzyme was obtained, and although the error in measurements is consequently higher, the inhibition by OLE of the crude and purified enzyme were not significantly different (*t*- test, 0.816, *p* > 0.05) (Fig. 3b). This indicates that other proteins present in the intestinal extract do not interfere with the estimation of inhibition. OLE also inhibited human maltase activity (Fig. 3c), with an IC_50_ value (Table 3) ~ twofold higher than that obtained for rat maltase.

OLE inhibited only weakly rat sucrase activity when tested up to a concentration of 2 mg oleuropein/ml (36.8 ± 1.6% inhibition). Sucrase activity in both rats and humans arises from a single enzyme, sucrase-isomaltase; a brush border enzyme which consists of two subunits. In comparison, OLE inhibited human sucrase activity dose-dependently (Fig. 3d) allowing an IC_50_ value to be estimated (Table 3).

The phenolic component of the OLE preparation used in vitro contains only extremely low levels of tyrosol and hydroxytyrosol. To ensure that these phenolics did not contribute to the observed inhibition we determined their content by HPLC (Table 1). Since the hydroxytyrosol concentration required to inhibit rat sucrase by 50% (i.e. IC_50_ value) was 0.0925 mg/ml, and the content of hydroxytyrosol in a 2 mg/ml Bonolive^®^ solution (0.8 mg oleuropein/ml) is 0.00013 mg/ml, we can safely conclude that the concentration of hydroxytyrosol is too low to contribute significantly to the observed inhibition. Likewise, rat maltase inhibition by hydroxytyrosol reached 9.7% at the highest concentration tested, 0.154 mg/ml (1 mM), which is much higher than the 0.00013 mg/ml present in a 2 mg/ml Bonolive^®^ solution. Tyrosol neither inhibited rat maltase nor sucrase at the highest concentration tested of 0.138 mg/ml (1 mM). The inhibitory activities reported here can therefore be confidently attributed to oleuropein, which constitutes > 80% of the phenolic composition.

### Effect of OLE on [^14^C(U)]-glucose transport across differentiated human Caco-2 cell monolayers

**Fig. 4:**
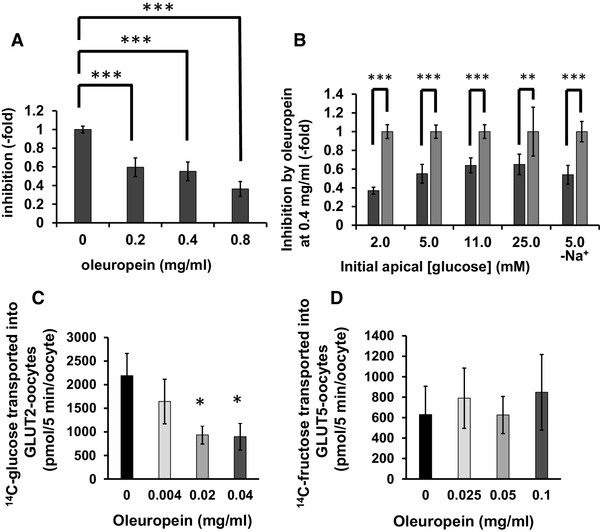
Effect of OLE on sugar transport. **a** Concentration dependence of OLE on transport of [^14^C(U)]-glucose (5 mM) across differentiated Caco-2/TC7 cell monolayers. ***p ≤ 0.001, compared to control with no OLE (*n* = 3 separate experiment with 6 replicates each). Error bars represent SD. **b** Effect of OLE (0.4 mg oleuropein /ml) on transport of [^14^C(U)]-glucose (5 mM) across differentiated Caco-2/TC7 cell monolayers at different apical [^14^C(U)]-glucose concentrations (*n* = 3 biological replicates with *n* = 6 wells per biological replicate; black bars). Error bars represent SD. **p ≤ 0.01, ***p ≤ 0.001 compared to control (grey bars) with no OLE. **c** Effect of OLE on glucose uptake by *Xenopus* oocytes expressing GLUT2. Two days post-cRNA microinjection, oocytes were incubated in 0.1 mM [^14^C(U)]-glucose with OLE for 5 min. Each data point represents the mean ± SEM of twelve replicates; IC_50_ = 0.012 ± 0.001 mg/ml. *p ≤ 0.05. **d** Effect of OLE on glucose uptake by *Xenopus* oocytes expressing GLUT5. One day post-cRNA microinjection, oocytes were incubated in 0.1 mM [^14^C(U)]-fructose with OLE for 5 min. Each data point represents the mean ± SEM of six replicates (18 oocytes)

OLE dose-dependently inhibited transport of [^14^C(U)]-glucose across differentiated Caco-2/TC7 cell monolayers (Fig. 4a), with IC_50_ ~ 0.5 mg oleuropein/ml. The inhibition of transport was observed over a range of glucose concentrations (2.5–25 mM) and was unaffected by the absence of sodium in the transport buffers (Fig. 4b).

### Effect of OLE on [^14^C(U)]-glucose and fructose transport into *Xenopus* oocytes expressing human GLUT2 or GLUT5

The lack of effect of sodium on inhibition by oleuropein suggested that GLUT transporters, and not sodium-dependent SGLT1, are the targets of oleuropein inhibition. We therefore determined if oleuropein could inhibit GLUT2 or GLUT5 expressed in *Xenopus* oocytes. OLE dose-dependently inhibited [^14^C(U)]-glucose transport by GLUT2 (Fig. 4c), but had no effect on [^14^C(U)]-fructose transport by GLUT5 (Fig. 4d).

### Effect of OLE on sucrose hydrolysis and transport by differentiated human Caco-2/TC7 cell monolayers

**Fig. 5:**
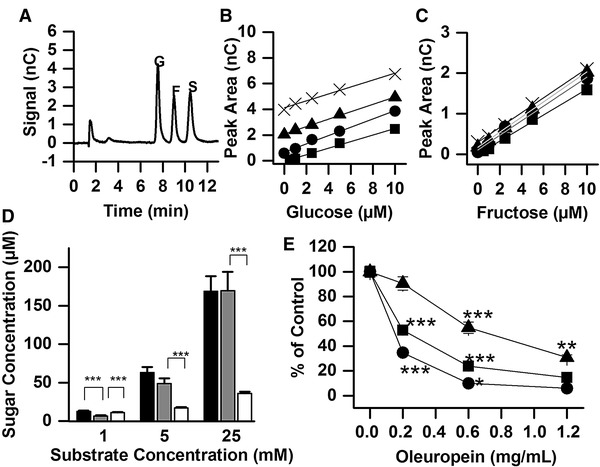
Effect of OLE on sucrose hydrolysis. **a** Representative trace of separation of glucose, fructose and sucrose at 5 μM (0.04 μl loaded; 0.2 pmol each loaded onto the column) on a Carbopac PA20 column at 0.008 ml/min by HPAE-PAD on an ICS-4000 system. **b, c** Standard curves used for quantification of glucose and fructose respectively in the presence of final concentrations of 0 (black square), 0.04 (black circle), 0.12 (black triangle) or 0.24 (cross symbol) mg oleuropein/mL. **d** Transport experiments in Caco-2/TC7 cells. After incubation with 1, 5 or 25 mM sucrose (60 min) in the apical compartment, apical fructose (black), apical glucose (grey) and basolateral glucose (white) were quantified by HPAE-PAD. Basolateral fructose concentration was < 5 µM within the timeframe of the experiment. **e** Concentration-dependence of OLE on sucrose hydrolysis and glucose transport by differentiated Caco-2/TC7 cell monolayers after incubation with 5 mM sucrose (60 min). Apical glucose (black square), basolateral glucose (black square) and apical fructose (black triangle) were quantified. Results are means ± SEM, transport experiments were performed with *n* = 3 biological replicates with *n* = 6 wells per biological replicate. ***p ≤ 0.001, **p ≤ 0.01, *p ≤ 0.05

To determine the fate of the sugars derived from sucrose hydrolysis and their subsequent transport we used a chromatographic method to detect and quantify sucrose, glucose and fructose (Fig. 5a–c). When sucrose was added to the apical side of differentiated Caco-2/TC7 differentiated cell monolayers, it was hydrolysed by sucrase on the brush border on the apical side of the cells into glucose and fructose in a concentration dependent manner (Fig. 5d). These sugars can then be absorbed by the cells and either used for energy, or transported to the basolateral compartment, and this led to the appearance of glucose in the basolateral compartment. Fructose was, however, below the limit of detection under these experimental conditions (concentration < 5 μM) (Fig. 5d). Addition of OLE to the apical compartment dose-dependently inhibited this process (Fig. 5e), owing to both inhibition of sucrase activity and of glucose transport (as shown in Figs. 3, 4).

### Effect of OLE on post-prandial glucose following consumption of bread by healthy volunteers (studies 1–5)

**Fig. 6:**
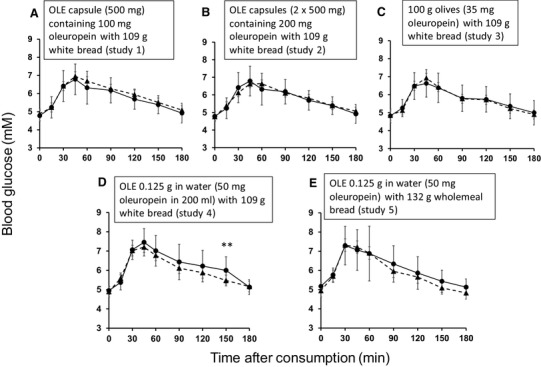
Effect of OLE or olives on postprandial blood glucose area under the curve during consumption of carbohydrates. Time dependence of blood glucose after consumption of control (black circle; with solid line) and test (black triangle; with dotted line) meals. **a** Study 1: double-blinded, randomised, crossover, placebo controlled in 24 healthy volunteers consuming bread (109 g containing 50 g carbohydrate) with OLE in capsules (500 mg, equivalent to 100 mg oleuropein). **b** Study 2: double-blinded, randomised, crossover, placebo controlled in 24 healthy volunteers consuming white bread (109 g containing 50 g carbohydrate) with OLE in capsules (2 × 500 mg, equivalent to 200 mg oleuropein). **c** Study 3: randomised, crossover, controlled study in 16 healthy volunteers consuming white bread (109 g containing 50 g carbohydrate) with 200 ml water with and without olives (100 g Kalamata olives containing 35 g oleuropein). **d** Study 4: randomised, crossover, controlled study on 10 healthy volunteers consuming white bread (109 g containing 50 g carbohydrate) with 200 ml water (control) or containing 125 mg dissolved OLE (50 mg oleuropein). **e** Study 5: randomised, crossover, controlled study in 10 volunteers consuming wholemeal bread (132 g containing 50 g carbohydrate) with 200 ml water (control) or containing 125 mg dissolved OLE (50 mg oleuropein). For additional details, see Table 2

We performed intervention studies on young, apparently healthy volunteers who consumed OLE in capsules or in solution together with bread (equivalent to 50 g available carbohydrate). In preliminary experiments, under conditions mimicking the stomach, the capsules released their contents within 3 min, and were completely dissolved within 5 min. Table 2 summarises the details of the intervention studies that were conducted. Post-prandial blood glucose was measured and the data is presented in Fig. 6. Consumption of OLE in capsules with white bread did not affect the post-prandial blood glucose concentrations over a 3 h period. Consumption of olives, or of OLE in solution, with white bread similarly produced no changes in blood glucose, and the effect was not changed if wholemeal bread was consumed.

### Effect of OLE on post-prandial glucose following consumption of sugars by healthy volunteers (studies 6–8)

**Fig. 7:**
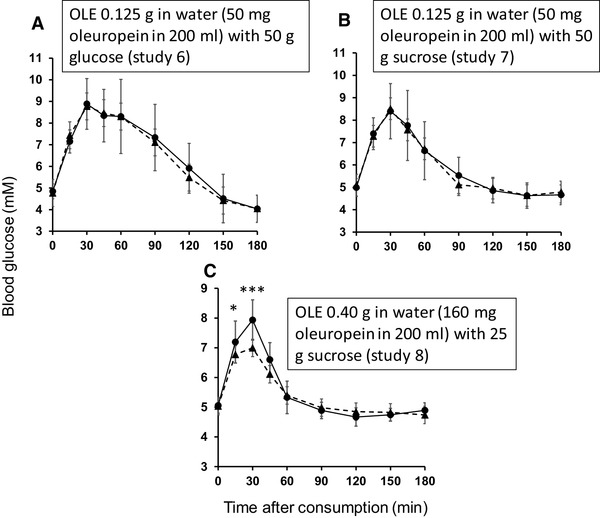
Effect of OLE on postprandial blood glucose area under the curve during consumption of sugars. Time dependence of blood glucose after consumption of control (black circle; with solid line) and test (black triangle; with dotted line) meals. **a** Study 6: randomised, crossover, controlled study on 10 healthy volunteers consuming glucose (50 g) with 200 ml water (control) or containing 125 mg dissolved OLE (50 mg oleuropein). **b** Study 7: randomised, crossover, controlled study on 10 healthy volunteers consuming sucrose (50 g) with 200 ml water (control) or containing 125 mg dissolved OLE (50 mg oleuropein). **c** Study 8: randomised, crossover, controlled study on 10 healthy volunteers consuming sucrose (25 g) with 250 ml water (control) or containing 400 mg dissolved OLE (160 mg oleuropein). For additional details, see Table 3

**Fig. 8:**
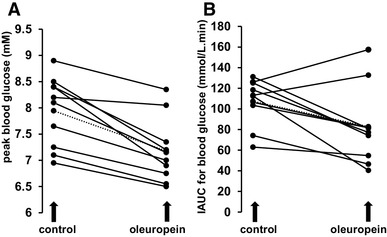
Inter-individual differences in responses to sucrose and oleuropein. Randomised, crossover, controlled study on 10 healthy volunteers consuming sucrose (25 g) with 250 ml water (control) (**a**) or 250 ml water containing 400 mg dissolved OLE (160 mg oleuropein) (**b**), indicating changes in IAUC for each volunteer by linked data points from control to treatment, with mean of all data shown as dotted line

**Fig. 9:**
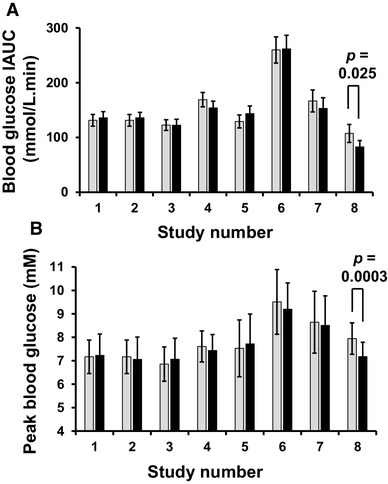
Summary of IAUC and peak blood glucose from intervention studies. Studies are numbered 1–9, and full details are given in Table 2

We performed intervention studies on young, apparently healthy volunteers who consumed OLE in solution together with glucose or sucrose (Table 2). Post-prandial glucose was measured and resulting curves are shown in Fig. 7. At the lower dose of sucrose and higher dose of OLE, a highly significant decrease (*p* < 0.0002) in peak glucose was observed in all individuals (Fig. 8). There was also a significant decrease in IAUC with OLE consumption (*p* = 0.025). When the dose of OLE was lower and given with 50 g glucose or sucrose, no significant effect was observed on post-prandial blood glucose. The changes in all intervention studies are summarised in Fig. 9.

## Discussion

Studies on rodents have shown effects of oleuropein on glucose metabolism and diabetes risk. For example, oleuropein (intraperitoneal, 5 mg/kg) significantly decreased serum glucose in streptozotocin-induced diabetic rats [28], and oral oleuropein attenuated hyperglycaemia and impaired glucose tolerance in the type 2 diabetes model Tsumura Suzuki Obese Diabetes mice with no effect on obesity [6]. In diabetic-hypertensive male Sprague–Dawley rats receiving oleuropein (20, 40, or 60 mg/kg/day), blood pressure and blood glucose, as well as infarct size, were significantly improved [29]. In C57BL/6 mice fed a 60% high-fat diet to generate non-alcoholic steatohepatitis, oleuropein (0.05% diet) over 6 months improved HOMA-IR and leptin levels [8], while serum glucose and cholesterol levels in alloxan-induced diabetic rats were decreased by oleuropein (8 and 16 mg/kg body weight) after 4 weeks [30]. In humans, OLE attenuated the digital volume pulse-stiffness index [9], and lowered HbA1c and fasting plasma insulin [11], but was not effective against oxidative damage markers [31] nor when combined with green coffee bean extract and beetroot powder [10].

**Fig. 10:**
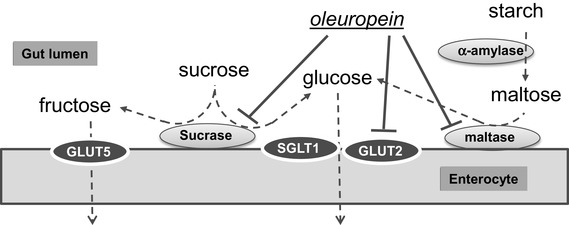
Mechanism of action of oleuropein on sucrose hydrolysis and subsequent sugar transport. Digestion of starch, maltose and sucrose in the gut lumen and transport of glucose across the intestinal barrier. The arrows indicate the sites where oleuropein exhibits inhibition according to our data

Several mechanisms could contribute to the protective effect(s) of dietary oleuropein [32, 33]. One mechanism to lower risk of developing type 2 diabetes is by consumption of low glycaemic index foods [34], which have significant health benefits when compared to diets rich in rapidly absorbable sugars [34]. Polyphenols may reduce post-prandial glycaemia [35], while any food components able to slow down carbohydrate digestion and glucose absorption across the small intestine blunting post-prandial blood glucose spikes could protect against development of type 2 diabetes [36]. The ability of acarbose to inhibit carbohydrate digestion is harnessed in the clinical setting [37] for diabetes management [38]. Some natural products and certain foods [13, 39–41] can act as acarbose-mimetics. As evidenced in healthy human volunteers, fruit pastes [19], coffee [42], green tea [43] and black tea [44] all modified some aspects of the glycaemic response. Given the wealth of evidence from animal studies, we decided to test oleuropein for its potential against developing type 2 diabetes through inhibition of carbohydrate digestion and sugar absorption. We first employed relevant in vitro human enzymic systems, and then explored whether the observed effects following a single dose were sufficient to potentiate an in vivo effect in healthy volunteers (Fig. 10).

Starch and maltodextrin(s) are digested by α-amylase, followed by maltase. The latter can arise from sucrase-isomaltase or maltase-glucoamylase, both located in the enterocyte brush border. Sucrose is digested only by the sucrase subunit of sucrase-isomaltase. The resulting sugars are absorbed by transport across the enterocytes into the blood stream by glucose transporters, predominantly GLUT2 and SGLT1 in the intestine, or by fructose transporters, mainly GLUT2 and GLUT5 (Fig. 10). We initially tested these sites for potential inhibition by OLE in vitro. Inhibition of human salivary α-amylase by OLE was relatively weak compared to acarbose and EGCG [21]. Inhibition of human and rat maltase activities by OLE were more potent than inhibition of α-amylase. OLE significantly inhibited transport of glucose by GLUT2 in both differentiated Caco-2/TC7 cell monolayers and into *Xenopus* oocytes expressing the human transporter. When the effectiveness of these in vitro results to reflect post prandial sugar metabolism in volunteers in vivo was tested, there was no effect following consumption of several different forms and doses up to 160 mg of OLE with bread (white or wholemeal), indicating that the efficacy of OLE on α-amylase is not sufficient to inhibit bread digestion. However, the combined action of OLE on sucrase and on glucose transport is sufficient to produce an attenuation of sucrose digestion and on appearance of glucose in the blood, but only at the lower dose of sucrose and higher dose of OLE.

Our in vitro data predict that oleuropein would not be modified in the small intestine, but could be absorbed or reach the colon intact where it would be metabolised by gut microbiota. This metabolism involves deglycosylation (Fig. 1) and conversion to lower molecular weight phenolics such as hydroxytyrosol [45, 46].

Since the effects reported here on carbohydrate digestion and sugar absorption are modest, it is likely that the dietary anti-diabetic effect of oleuropein is not through modulation of post prandial sugar metabolism. Instead, beneficial bioactivities could be prompted by the modest absorption of intact oleuropein, or by the well absorbed lower molecular weight phenolics such as hydroxytyrosol, produced from gut microbiota-catalysed catabolism of oleuropein [47]. Such a hypothesis underlines the potential of gut microbial metabolism to indirectly affect metabolic health and warrants further research. The effects on sugar absorption reported here could be exploited through the design of supplements or functional foods [48] but it is unlikely they can account for the anti-diabetic effects of oleuropein [49], as naturally present in olives and olive oil [1] from the diet.

## Abbreviations

OLE: Olive leaf extract
IAUC: Incremental area under the curve
GLUT2: Solute carrier family 2, member 2 (SLC2A2)
GLUT5: Solute carrier family 2, member 5 (SLC2A5)
DMSO: Dimethyl sulfoxide
